# Phenotyping floral traits and essential oil profiling revealed considerable variations in clonal selections of damask rose (*Rosa damascena* Mill.)

**DOI:** 10.1038/s41598-023-34972-5

**Published:** 2023-05-19

**Authors:** Ajay Kumar, Rahul Dev Gautam, Satbeer Singh, Ramesh Chauhan, Manish Kumar, Dinesh Kumar, Ashok Kumar, Sanatsujat Singh

**Affiliations:** 1 Academy of Scientific and Innovative Research, Ghaziabad, Uttar Pradesh 201002 India; 2 Agro Technology Division, Council of Scientific and Industrial Research—Institute of Himalayan Bioresource Technology, Post Box No. 6, Palampur, Himachal Pradesh 176 061 India; 3 Chemical Technology Division, Council of Scientific and Industrial Research—Institute of Himalayan Bioresource Technology, Post Box No. 6, Palampur, Himachal Pradesh 176 061 India

**Keywords:** Plant breeding, Plant sciences, Secondary metabolism

## Abstract

Damask rose (*Rosa damascena* Mill.) is a high-value aromatic plant species belonging to the family Rosaceae. It is being cultivated throughout the world for rose essential oil production. Besides its higher demand in the aromatic and cosmetic industry, the essential oil obtained has many pharmacological and cytotoxic activities. The primary concern of growers with the available varieties of damask rose is short flowering duration, low essential oil content and unstable yield. Thus, there is a requirement for developing new stable varieties with higher flower yield and essential oil content. The present study evaluated the variations in the flower yield parameters, essential oil content, and essential oil compounds in different clonal selections of damask rose. These clonal selections have been developed through a half-sib progeny approach from commercially available varieties 'Jwala' and 'Himroz.' The fresh flower yield varied from 629.57 to 965.7 g per plant, while the essential oil content ranged from 0.030–0.045% among the clonal selections. The essential oil profiling via gas chromatography–mass spectrometry revealed significant variations in the essential oil compounds. Acyclic monoterpene alcohols citronellol (20.35–44.75%) and geraniol (15.63–27.76%) were highest, followed by long-chain hydrocarbons, i.e., nonadecane (13.02–28.78%). The clonal selection CSIR-IHBT-RD-04 was unique in terms of the highest citronellol content (44.75%) and citronellol/geraniol (C/G) ratio of 1.93%. This selection has the potential use as a parental line in future genetic improvement programs of damask rose to achieve higher yield and better quality of rose essential oil.

## Introduction

*Rosa damascena* Mill., also known as **“**damask rose," is a valuable aromatic member of the Rosaceae family. It belongs to the genus *Rosa*, comprising nearly 200 species and about 1800 cultivars^1^. It is an erect, perennial, hermaphrodite shrub possessing multiple green prickly stems up to 2 m in height and compound leaves with oval serrated leaflets^2^. Flowering in damask rose occurs during the onset of the summer season and continues for 30–35 days^3^. The species originated in the Damascus region of Asia Minor and occupied one of the most important positions as an aromatic plant for extracting essential oil. It is suitable for cultivation in sub-tropical and temperate zones of the northern hemisphere^4^.

The major cultivating countries of the damask rose worldwide are China, Bulgaria, France, Italy, Turkey, Iran, Morocco, Russia, USA and India^1,5^. The production of its essential oil is nearly 4.5 tons/year globally, where Turkey and Bulgaria contribute up to 90% of the total output^6,7^. In recent reports, the global rose oil market value was ~ 279 million USD in 2018, which will increase in the near future^7^. The commercial cultivation of damask rose in India dates back to Mughal times. Presently, the damask rose has cultivation in the northern regions, e.g., Himachal Pradesh, Jammu and Kashmir, Rajasthan, Haryana, Uttar Pradesh and Punjab, with an annual production of 200 kg of essential oil^5,8^. The temperate climatic conditions and suitable soil in the State of Himachal Pradesh are best suitable for cultivating perfumery roses^9^.

Rose-water (hydrosol), essential oil, concrete and absolute are the major industrial products obtained from damask rose. These can be obtained through hydro-distillation and solvent extraction processes^1,10^. Damask rose is considered best and cultivated worldwide due to the superior quality of its essential oil^11^. The essential oil of damask rose has wide use in manufacturing perfumes, colognes and cosmetics, while rose water, which is the by-product, has extensive demand in the flavoring industry^12^. The other products obtained from damask rose are "gulkand" and orthodox tea^13^. The essential oil of damask rose is the most expensive in the global market due to low oil content and high demand^1^. Various investigations have been conducted to evaluate the essential oil composition of damask rose via GC/MS methods^1,5,7,14,15^. The major compounds identified in the essential oil of rose are acyclic monoterpene alcohols and long-chain hydrocarbons^12^. The major essential oil compounds reported in rose oil are *β*-citronellol, nonadecane, geraniol, heneicosane and eugenol^1,7,16^. Although, the essential oil quality depends upon the relative content of the oil's Citronellol/Geraniol ratio (C/G Ratio)^1^. The essential oil obtained from damask rose is reported to have many pharmacological, cytotoxic and genotoxic activities^17,18^.

Damask rose is cultivated over a wide range of environmental conditions, and the essential oil quality varies for genotypes, time of flower harvest, stage of the crop, methods of distillation, and agronomic factors^1,8,19,20^. Damask rose (2n = 28; tetraploid) is a cross-pollinated perennial species propagated clonally through cuttings. It is considered an inter-specific hybrid which could have arisen from *R. gallica* × *R. moschata* for the “summer” damask rose or from *R. gallica* × *R. phoenicea* for the “autumn” damask rose group^21^. Different clonal types vary considerably for phenotypic and essential oil quality traits, and overall, these attributes have strengthened the prospects of damask rose as an industrially important crop. However, it did not assist in widening the genetic variability of the species as cultivation practices were mostly confined to identifying elite plants and their propagation for widespread cultivation. Presently, the major constraint in the available varieties of damask rose is the short flowering duration, low oil content and unstable yield over the locations and years. These issues need to be addressed through a sustainable breeding program to develop new stable varieties of damask rose with enhanced essential oil production. In the genetic improvement program of the damask rose, a critical appraisal of floral traits is a pre-requisite as flowers constitute the economic produce of the plant. Therefore, germplasm appraisal based on phenotypic floral variations and essential oil profiling is vital for improving productivity and selecting desirable variations for integration in the breeding program. The present study is an endeavor to organize the damask rose genetic resources based on phenotypic and chemotypic characteristics, select potential genotypes for cultivar development and identify genetically diverse clonal lines for future breeding.

## Material and methods

### Experimental material and location

The present investigations were conducted on four newly developed damask rose selections made in half-sib progeny lines. The lines were derived from commercial varieties 'Jwala' and 'Himroz.' The lines are being maintained clonally in the rose germplasm repository along with check varieties (Jwala and Himroz) at CSIR-Institute of Himalayan Bioresource Technology, Palampur (1320 m above mean sea level, 32°68'N, 76°38'E). The rose germplasm repository at CSIR-IHBT maintains different cultivated and wild Rosa species from India and worldover, which were introduced through the Indian Council of Agricultural Research–National Bureau of Plant Genetic Resources (ICAR-NBPGR), Regional station at Phagli, Shimla (Himachal Pradesh), India. The location is the mid-hills zone (Zone-II) of Himachal Pradesh (India), which represents sub temperate and humid climate with a mean annual rainfall of ~ 2500 mm, mainly during the monsoon season (July–September). The rose germplasm repository at CSIR-IHBT maintains different cultivated and wild Rosa species from India and the world. The study was carried out on five-year-old plants of each clonal line over two consecutive years (2021 and 2022). A basal fertilizer dose calculated as 120 kg nitrogen (N), 60 kg phosphorus (P), and 40 kg potassium (K) per ha was applied during both years. All the agronomic practices were followed as per the recommendations. The experiment was set up in Randomized Complete Block Design (RCBD), having 1.5 m of plant spacing between rows and 0.75 m within rows. The number of replications for each clonal line is four. It is also to confirm that all methods were performed in accordance with the relevant guidelines and regulations.

### Observation for yield-related floral traits

**Figure 1:**
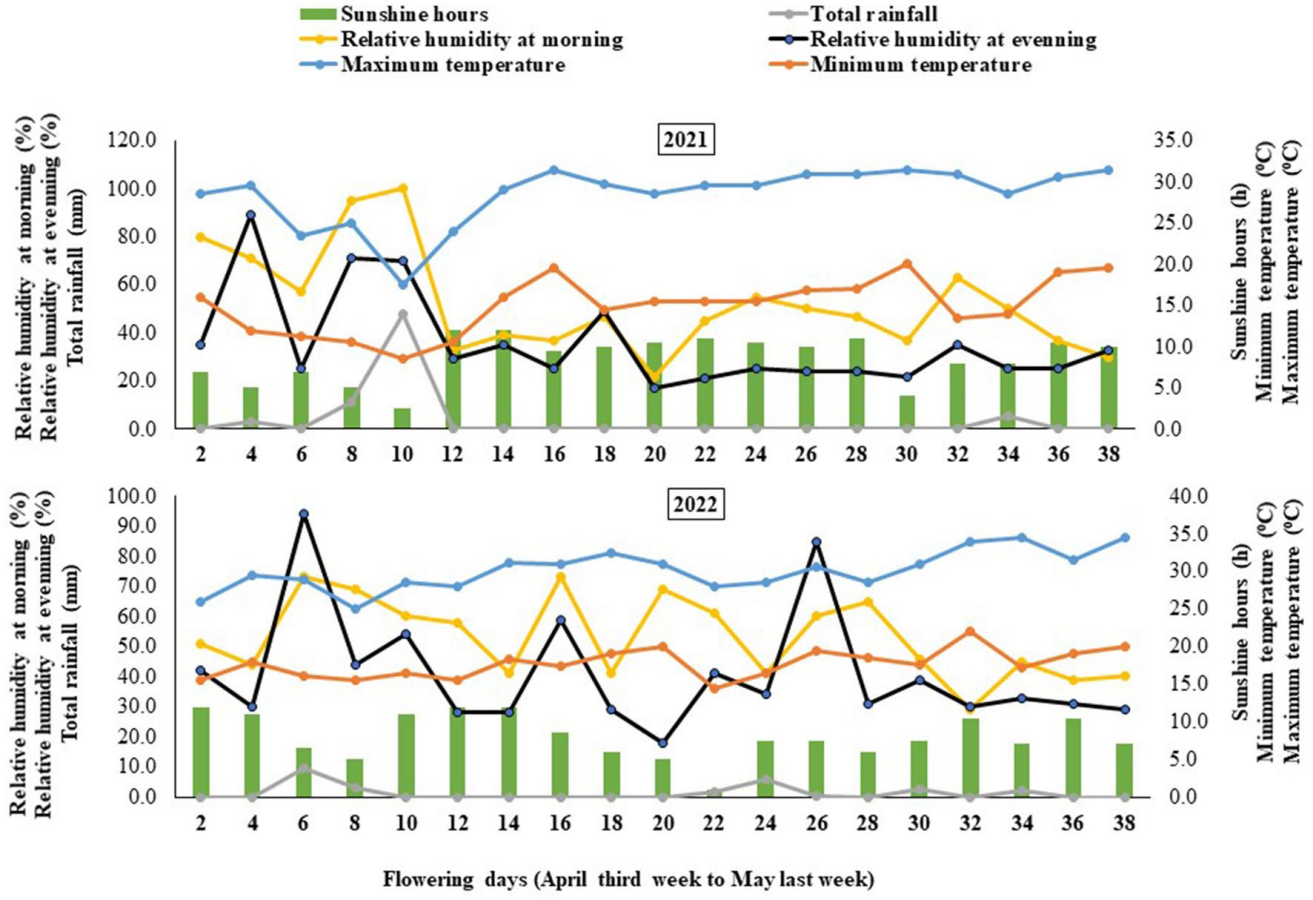
Meteorological data during flowering period of damask rose (April third week to May last week) for two growing seasons (2021 and 2022).

Data was recorded on four random competitive plants per clonal line in each replication. These plants were tagged, and observations were repeated during the second year. The morphological parameters recorded in both years were the number of flower-bearing shoots/plant, flower weight (g), flower diameter (cm), petal number, petal length (cm), petal width (cm), petal thickness (mm), petal weight/flower (g), flower frequency/plant/day, flower number/plant, flower yield/plant, number of flowering days. Data were recorded daily during the flowering period (April third week to May last week). The weather data during the flowering period of the damask rose for both the experimental years (2021 and 2022) is given in Fig. 1.

### Essential oil extraction

The flowers were picked manually during morning hours (6:00–9:00 am) to avoid loss of aroma compounds for both years. Fresh flowers (1 kg) of each clonal line were harvested, and essential oil extraction was done by hydro-distillation for four hours (in triplicate), using Clevenger-type apparatus of five liters distillation system. The ratio of flower to water used for extraction was 1:2 (w/v). The essential oil obtained from each sample was measured, and oil content (w/w) was depicted in percentage (%) on a fresh weight basis. The moisture content in essential oil was removed using sodium sulfate (anhydrous). The essential oil is collected in a glass vial and kept in a refrigerator (4–6 °C) till further chemical characterization. After that, gas chromatography–flame ionization detector (GC–FID) and GC–mass spectrometry (GC–MS) analysis were used for the chemical characterization of essential oil compounds present in rose oil.

### GC–MS/GC analysis of essential oil

GC–MS characterization of damask rose essential oil was performed using a Shimadzu GC–MS QP2010 gas chromatograph attached to a flame ionization detector (FID). The essential oil was analyzed over SH-RX-5Si/MS capillary column, Shimadzu Asia Pacific, USA (30 m × 0.25 mm × 0.25 μm film thickness) attached to the gas chromatograph. The GC–MS analysis was carried out with the same conditions reported earlier^22–24^. The retention indices (RI) for all the chemical compounds were calculated using homologous series of n-alkanes C9–C24 (SUPELCO, Sigma-Aldrich). The retention indices were calculated for every GC–MS spectra peak to identify the compounds. The calculated retention indexes were compared with Adams tabulated indexes^25^ stored in the NIST-mass spectral database^26^. After identifying the essential oil compounds, the next step was the quantification performed through GC analysis. The GC analysis was carried out using Shimadzu GC 2010 gas chromatograph attached to a flame ionization detector (FID). The analysis was performed on the same capillary column described above. The instrument was operated with the same conditions reported earlier^22–24^ . Finally, individual compounds were quantified using the peak area percentage of the chromatogram. Also, the mass spectral fragment patterns of the chemical compounds were compared with those reported in the literature.

### Statistical analysis

The phenotypic data of floral traits and damask rose clonal lines yield were recorded for two consecutive years. The analysis of variance (ANOVA) was performed to test the performance of clonal lines during both years. The Variations among clonal lines were determined using the F-test (comparing genotypes' mean with check varieties). Data for the morphological traits were analyzed using multivariate clustering following Euclidean similarity co-efficient with Past 1.40 software^27^. The Eigenvalues of characters loading were calculated to find out the effect of characters on the clustering. Principal component analysis was done to identify key characters which differentiate the clonal lines into distinct groups. The correlation studies were executed to explore the relationship between floral traits using the Pearson correlation matrix. The correlation coefficient (r) for different essential oil compounds was calculated using OP STAT^28^, and the matrix was prepared using Past 1.40 software.

## Results and discussion

### Variations in the yield-related floral traits

Significant variations were observed among the damask rose clonal lines for floral traits studied during both years. Based on the F-value significant differences among lines were obtained for flower-bearing shoots, flower frequency/plant/day, flower number per plant and flower yield per plant (Table 1). The number of flowers per plant is the most important component determining flower yield per plant^29^. A high level of genetic diversity has been reported earlier^2^ based on morphological traits among and within the *Rosa* species from the western Himalayan region. The phenotypic variability obtained in the present study has a genetic basis, and the studied traits help differentiate the different clonal lines of damask rose. These variations in flower characteristics might be due to the segregation of the alleles at heterozygous loci. Similar phenotypic variations were identified earlier among the accessions for commercially important morphological traits in damask rose germplasm to select superior accessions^16,30^. Mahajan and Pal, 2020^12^ have also reported significant variations in the number of flowers and flower yield while studying the effect of seasonal variations on floral traits in damask rose.

**Table 1:** ANOVA test for the differences in floral traits among six clonal selections of damask rose based on the mean values of data for both the years (2021 and 2022).

S. No	Floral traits	Variance		F value		CV (%)	
		2021	2022	2021	2022	2021	2022
1	Flower weight	0.65	0.28	2.35	2.93	11.66	6.58
2	Flower diameter	0.91	0.70	2.09	2.01	8.20	7.01
3	Petal Number	17.87	25.48	0.38	2.23	16.42	8.58
4	Petal length	0.37	0.04	1.51	0.24	11.77	9.70
5	Petal width	0.32	0.05	0.11	0.30	50.41	10.92
6	Petal thickness	0.0001	0.00078	0.45	3.02	8.25	7.97
7	Petal weight/flower	0.33	0.29	1.64	2.09	14.86	11.90
8	Flower number/plant	3135.67	4509.27	10.28*	68.18*	9.92	4.88
9	Flower yield/plant	57,954.77	54,740.28	363.62*	711.62*	1.61	1.12
10	Number of flowering days	1.04	0.97	0.51	1.07	3.88	2.55
11	Flower bearing shoots	13.64	15.90	22.84*	31.80*	10.36	9.75
12	Flower frequency/plant/day	2.16	3.14	10.24*	56.38*	9.91	5.38

*significant at *P* ≤ 0.05.

Similarly, Zeynali and co-workers, 2009^29^ reported flowers per plant as an important component controlling flower yield per plant. Identifying new variations in damask rose is important in enriching the damask rose germplasm resources and their use in future genetic improvement programs. Multivariate clustering of the phenotypic data based on quantitative floral traits (flower-bearing shoots, flower number per plant and flower yield per plant) differentiated the clonal lines into distinct phenotypic groups highlighting the degree of variation within each group. It is suggested that the phenotypic stability of desirable traits in a population, line or accession set is important for further utilization in hybridization programs to achieve genetic improvement^31^.

### Principal component analysis

**Figure 2:**
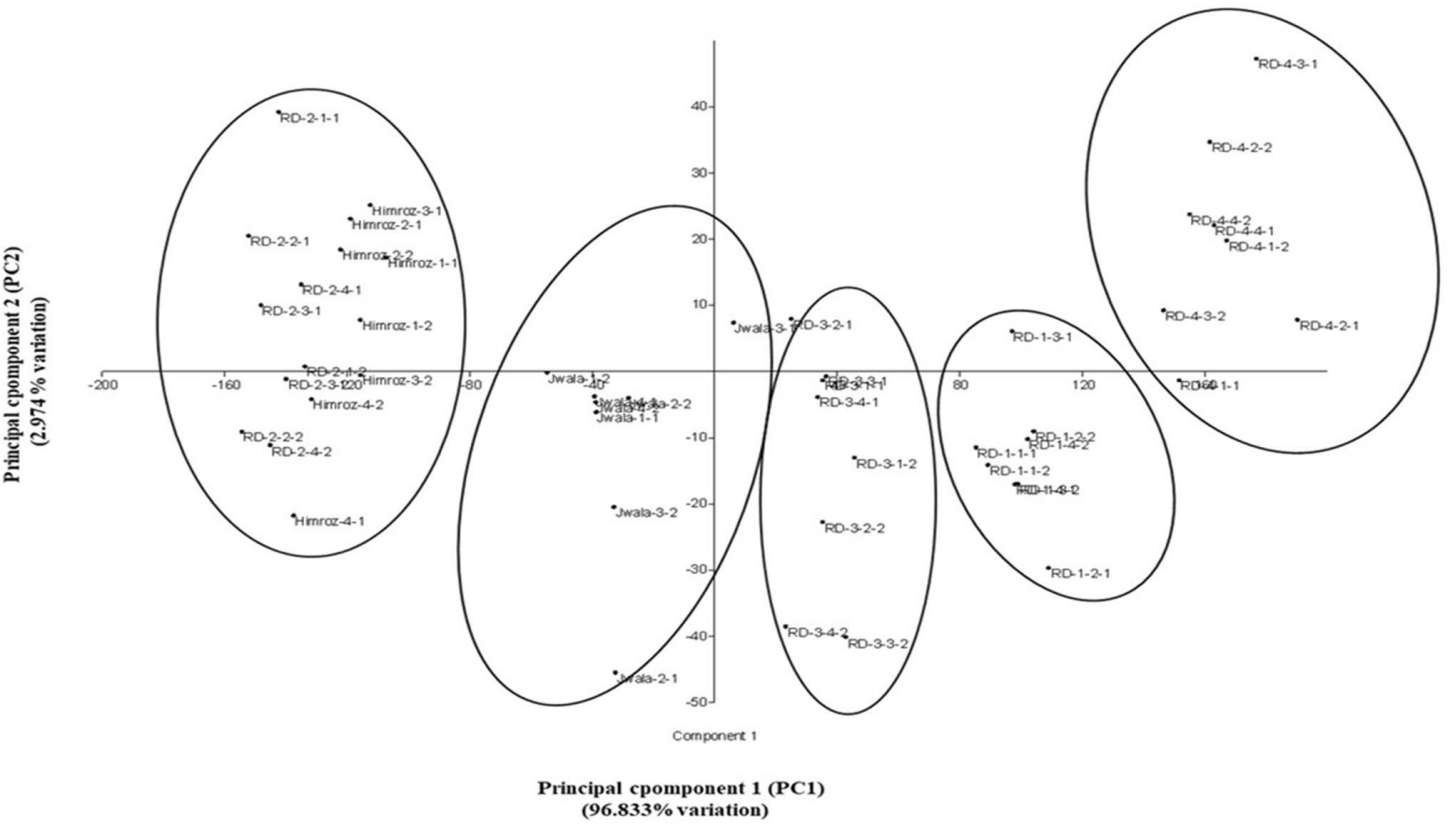
Grouping of the clonal selections through variance–covariance matrix scatter plot of principal component traits.

Principal component analysis (PCA) was performed to explore the relationship among the different floral traits to identify key principal traits based on the highest eigenvalue (Table 2). Based on PCA analysis using pooled data from two years, the flower-bearing shoots were the key principal component (PC1), explaining 96.833% of the variance. In contrast, flower weight was the 2nd principal component (PC2), explaining 2.974% of variance that influenced the differentiation and clustering of clonal lines, whereas all other floral traits had low eigenvalue loadings. However, using variance–covariance matrix scatter plot of principal components, plants of clones CSIR-IHBT-RD-01, CSIR-IHBT-RD-03, CSIR-IHBT-RD-04 and Jwala grouped independently, while those of Himroz and CSIR-IHBT-RD-02 grouped in the same cluster (Fig. 2).

**Table 2:** Principal component analysis of the phenotypic traits based on Eigen value loading of floral traits.

S. No	Principal components	Eigen value	% variance
1	Flower bearing shoots	12,696.10	96.83
2	Flower weight	390.01	2.97
3	Flower diameter	22.72	0.17
4	Petal Number	1.20	0.009
5	Petal length	0.51	0.003
6	Petal width	0.31	0.002
7	Petal thickness	0.28	0.002
8	Petal weight/flower	0.09	0.0007
9	Flower number/plant	0.07	0.0005
10	Flower yield/plant	0.01	0.0001
11	Number of flowering days	0.008	0.00006
12	Flower frequency/plant/day	0.0001	0.000001

The maximum flower yield was obtained in clonal selection CSIR-IHBT-RD-04 (944.07 g/plant in the first year and 931.05 g/plant in the second year), followed by CSIR-IHBT-RD-01 (880.08 g/plant in 2021 and 881.19 g/plant in 2022), CSIR-IHBT-RD-03 (813.91 g/plant in 2021 and 824.48 g/plant in 2022), Jwala (759.52 g/plant in 2021 and 746.34 g/plant in 2022), Himroz (663.77 g/plant in 2021 and 662.9 g/plant in 2022) and CSIR-IHBT-RD-02 (636.88 g/plant in 2021 and 643.76 g/plant in 2022). Based on the F-value, the weight of the individual flower (3.56–6.34 g), flower diameter (6.4–9.7 cm), petal number (30–54), petal length (3–4.9 cm), petal width (2.3–5.0 cm), petal thickness (0.15–0.25 mm), the weight of petals per flower (2.38–4.62 gm) and the number of flowering days (34–38) showed non-significant variations among the clones. Whereas significant variations were observed for flower number per plant (120–257), flower yield per plant (629.57–965.7 g), flower-bearing shoots (5–12) and flower frequency/plant/day (3.15–6.76) in both the years (Table 1).

**Figure 3:**
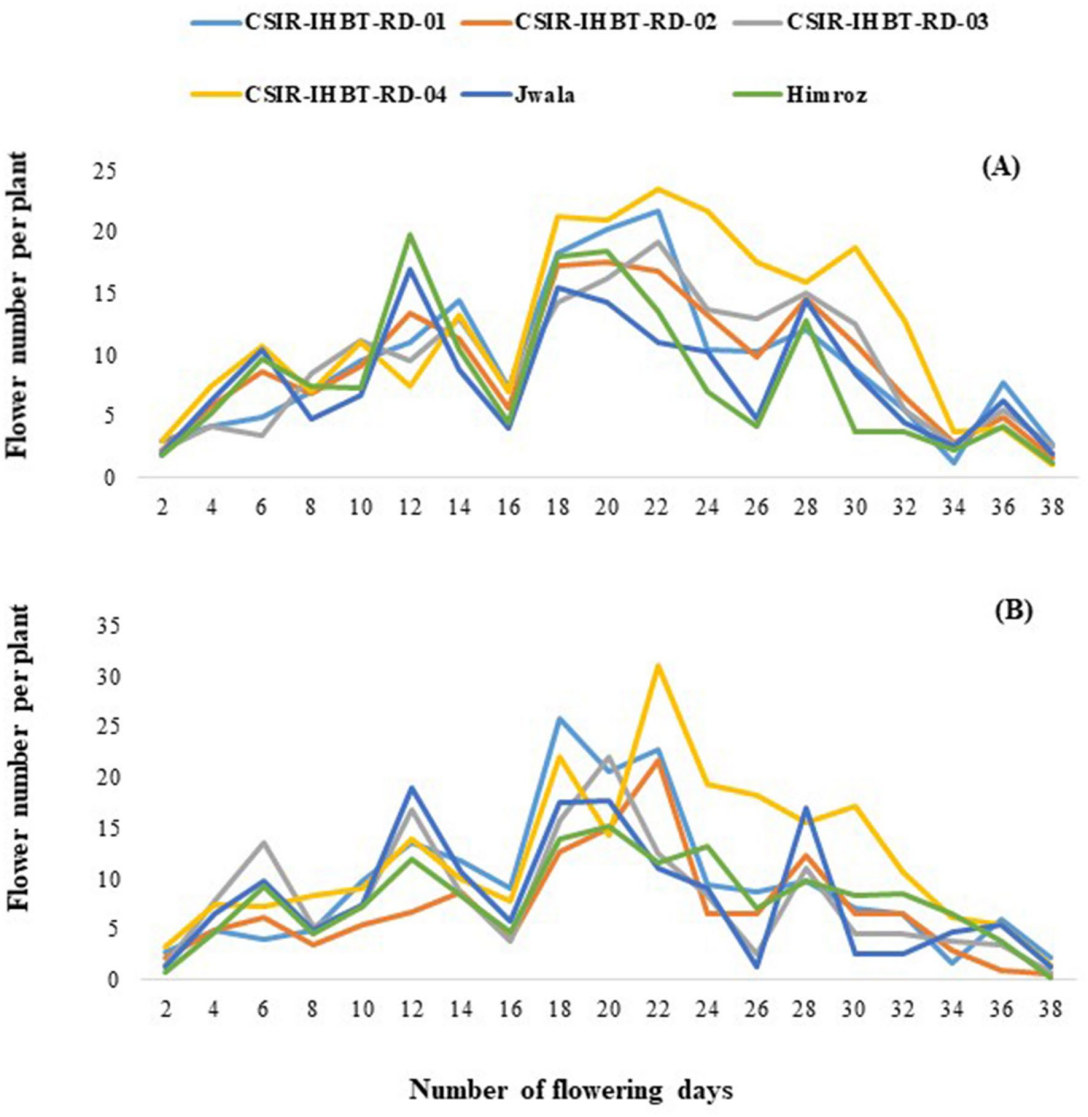
Variations of mean flower frequency per plant on daily basis, (**A**) during flowering season, 2021 and (**B**) during flowering season, 2022.

Overall, based on the mean value of both studied years, CSIR-IHBT-RD-04 (937.56 g/plant) was superior, followed by CSIR-IHBT-RD-01 (880.63 g/plant) and check variety Jwala (752.93 g/plant). Check variety Himroz (663.33 g/plant) was inferior to CSIR-IHBT-RD-03 (819.19 g/plant) but performed better than CSIR-IHBT-RD-02 (640.32 g/plant). Based on mean flower frequency per plant daily, peaks were observed almost weekly for the clonal lines in both years. The mean flower frequency/plant/day was the least at the initiation and fag end of the flowering season. It reached a maximum after about 20 days of flower initiation during both years (maximum flower frequency/plant/day) based on the pooled average of clonal lines as 17.95 in 2021 and 18.42 in 2022 (Fig. 3).

In the case of CSIR-IHBT-RD-04, there was consistently higher flower frequency/plant/day from 22–32 days of the flowering period compared to other clonal lines, which differentiated the line based on its phenology. An earlier report based on principle component analysis suggested that parents with a higher fresh weight of Flower, number of petals per flower and bud width can be used for hybridization during the genetic improvement program of damask rose^29^.

### Correlation and regression analysis

Correlation studies were performed based on the two-year pooled data of the floral traits to identify significant variation. Based on the correlation matrix (Table 3) for the floral traits studied, Flower bearing shoots had a significantly high correlation with flower number per plant (r = 0.946), flower yield/plant (r = 0.775) and flower frequency/plant/day (r = 0.940). In contrast, Flower bearing shoots showed a moderate negative correlation with flower weight (r =  − 0.504). Flower weight shows a moderate positive correlation with flower diameter (r = 0.401), petal thickness (r = 0.413), petal weight (r = 0.627) and a moderate negative correlation with flower/frequency/plant/day (r =  − 0.580). Flower diameter and petal length show a moderate correlation (r = 0.434), while petal length and width also show a moderate correlation (r = 0.489). Petal width positively correlates with petal thickness (r = 0.521). Petal thickness negatively correlates with flower number/plant (r =  − 0.427) and flower frequency/plant/day (r =  − 0.409). A significantly high positive correlation was obtained between flower number and flower yield (r = 0.770) and flower frequency (r = 0.987). Flower yield per plant was highly correlated with flower frequency/plant/day (r = 0.770). Pal and Mahajan (2017)^32^ reported a similar observation based on PCA of the floral traits where flower yield and weight were significantly correlated with the number of flowers and petal number, respectively.

**Table 3:** Correlation matrix for the floral traits in germplasm lines of damask rose based on the mean values of pooled data of two years (2021 and 2022).

	Flower diameter	Petal Number	Petal length	Petal width	Petal thickness	Petal weight/flower	Flower number/plant	Flower yield/plant	Number of flowering days	Flower bearing shoots	Flower frequency/plant/day
Flower weight	0.401	0.179	0.159	0.400	0.413	0.628	− 0.593	0.039	− 0.247	− 0.505	− 0.580
Flower diameter		0.030	0.434	0.170	0.362	0.099	− 0.321	− 0.077	0.035	− 0.318	− 0.319
Petal Number			0.081	− 0.040	− 0.240	0.180	0.138	0.307	− 0.247	0.114	0.156
Petal length				0.489	0.372	0.025	− 0.168	− 0.071	− 0.100	− 0.204	− 0.193
Petal width					0.521	0.329	− 0.161	0.107	− 0.021	− 0.115	− 0.153
Petal thickness						0.116	− 0.428	− 0.182	0.027	− 0.342	− 0.410
Petal weight/flower							− 0.198	0.210	− 0.260	− 0.077	− 0.175
Flower number/plant								0.771*	0.349	0.956*	0.987*
Flower yield/plant									0.259	0.776*	0.770*
Number of flowering days										0.321	0.350
Flower bearing shoots											0.941*

*significant at *P* ≤ 0.05.

**Figure 4:**
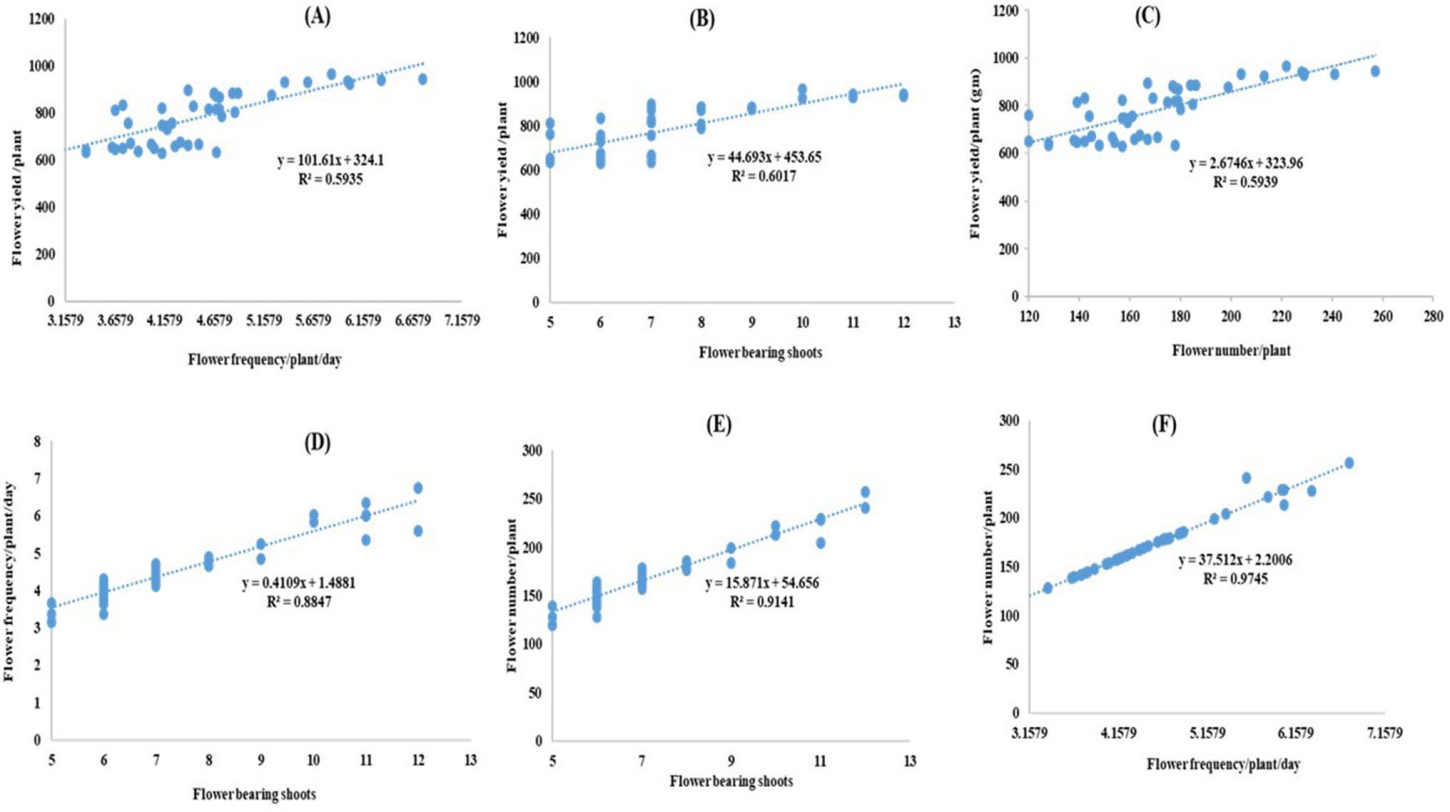
Regression plots for floral traits where independent variables lie towards X-axis and dependent variable lies towards Y-axis.

A high correlation among traits indicates a strong association among the traits, whereby one trait influences the expression of the other. Accordingly, a regression equation was established between independent variables, i.e., flower frequency/plant/day, flower-bearing shoots and flower number/plant) and dependent variable, i.e., flower yield per plant, to establish the association based on a second-degree polynomial relationship. Figure 4A, B and C displayed a comparatively low statistical correlation between these independent variables to the flower yield with a coefficient of determination (*R*^*2*^) ranging from 0.59 to 0.60. The inter-relationships among flower frequency/plant/day, flower-bearing shoots and flower number/plant were also tested using regression analysis.

The flower frequency/plant/day and flower-bearing shoots (*R*^*2*^ = 0.88, Fig. 4D) showed better relationships among each other. Flower number/plant showed a comparatively higher association with Flower bearing shoots (*R*^*2*^ = 0.91, Fig. 4E). In comparison, the flower number/plant showed a stronger second-degree polynomial relationship (y = 37.512x + 2.2006, *R*^*2*^ = 0.97, Fig. 4F) with flower frequency/plant/day. Flower yield in damask rose is an economically crucial trait after essential oil quality and accurate floral phenotyping is critical to identify potential selections and maximize production.

### Essential oil yield (w/w %)

**Figure 5:**
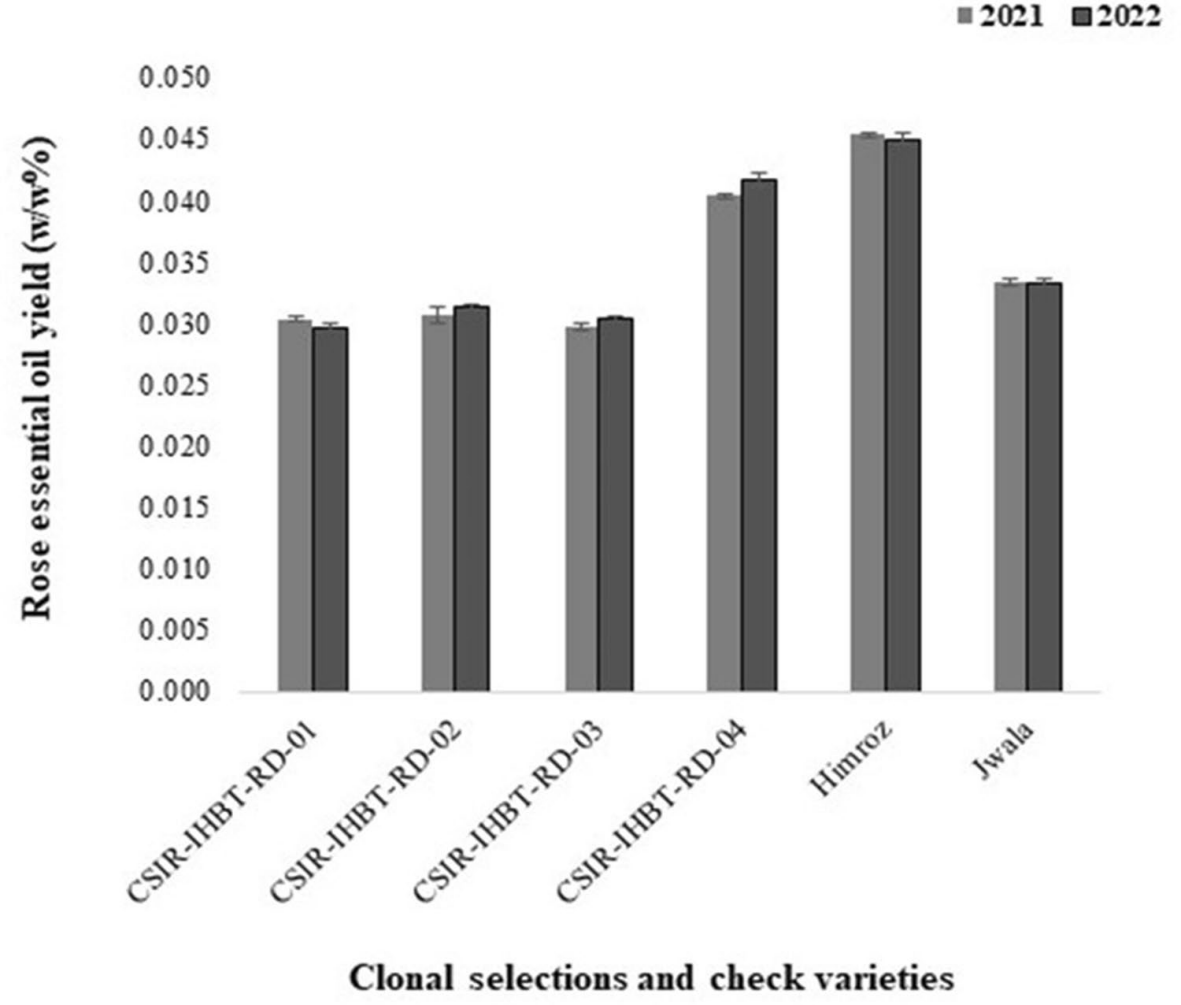
Variations in essential oil yield of damask rose clonal selections and check varieties during 2021 and 2022.

**Figure 6:**
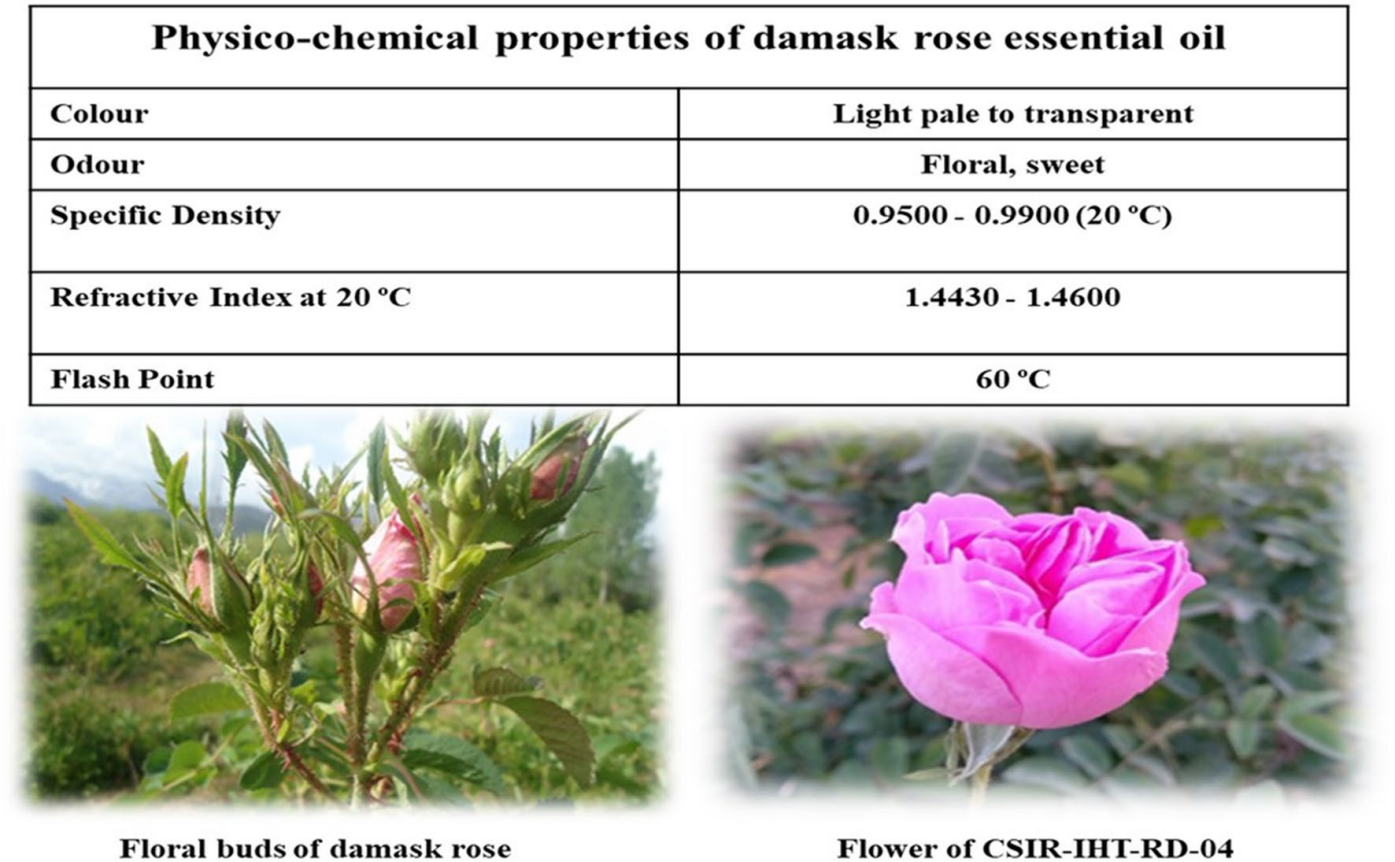
Physico-chemical properties of essential oil along with depiction of floral buds and flower of clonal selection CSIR-IHBT-RD-04.

The essential oil yield of the four clonal selections (CSIR-IHBT-RD-01 to CSIR-IHBT-RD-04) and two check varieties (Himroz and Jwala) maintained at CSIR-IHBT Palampur are depicted in Fig. 5. The essential oil content varies from 0.030 to 0.045% of the fresh flower weight in kilogram during both the experimental years (2021 and 2022). The important physico-chemical properties of the damask rose essential oil is depicted in Fig. 6. Based on the comparison of four selections in terms of rose oil yield, the clonal selection CSIR-IHBT-RD-04 showed a higher percentage of essential oil (0.040% in 2021 and 0.042% in 2022) compared to others clonal selections. However, a t-test using standard deviation shows significant variations in essential oil yield (0.45%) in the check variety Himroz for both years. Usually, the yield of essential oil in damask rose from the western Himalayas is reported to be 0.017 to 0.051^5^. However, through appropriate agronomical interventions, the essential oil content may reach a high of 0.056% under the acidic conditions of the western Himalayas^33^. In a recent study from Iran, the essential oil content was reported to be 0.03–0.04%^1^. The genetic architecture of the plant species might be another reason for the variation in essential oil content during the present study. Clonal selection has the advantage of maintaining the homogenous grade of essential oil for industrial use^24^. Accordingly, evaluating clonal lines is necessary for selecting superior clones with a higher essential oil yield for a specific region.

### GC–MS based essential oil profiling of clonal lines and check varieties indicates chemotypic distinctions

**Figure 7:**
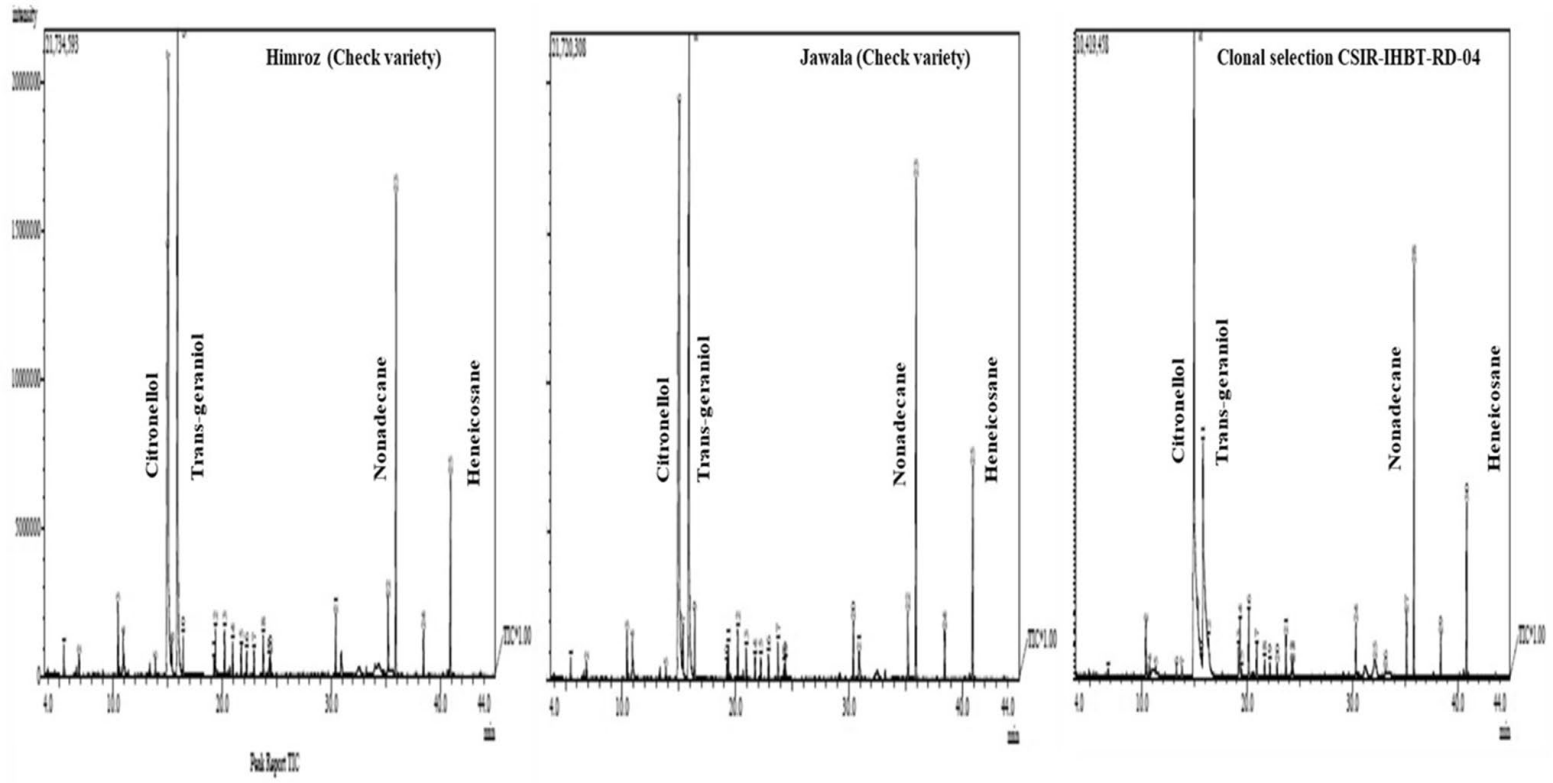
Representative GC–MS chromatogram of major compound of damask rose essential oil, where CSIR-IHBT-RD-04 is showing chemotypic distinction from check varieties “Himroz” and “Jwala”.

A comparative study of essential oil composition was carried out by gas chromatography-mass spectrophotometry (GC–MS) to understand the chemotypic distinctions with respect to essential oil composition in four clonal lines (CSIR-IHBT-RD-01 to CSIR-IHBT-RD-04), and two check varieties (Himroz and Jwala) of damask rose. Hydro distillation of fresh flowers led to obtaining uncolored to yellowish essential oil. Overall, twenty-six compounds were identified in the essential oil via GC–MS analysis, which accounted for 97.04 to 99.48% of the total essential oil profile. The essential oil components were grouped into oxygenated monoterpenes (36.62 to 70.05%), oxygenated sesquiterpenes (2.80 to 6.57%), sesquiterpene hydrocarbon (2.63 to 6.40%) and aliphatic hydrocarbons (19.94 to 55.68%). The retention time and indices of all the essential compounds are summarized in Table 4. The analysis of composition data suggested that the oxygenated monoterpenes and aliphatic hydrocarbon were the major fractions in the essential oil. The representative GC–MS chromatogram of the major compound of damask rose essential oil is given in Fig. 7.

**Table 4:** GC–MS profiling based variations of essential oil compounds in clonally propagated lines and two check varieties of damask rose during 2021 and 2022.

S. No	Compound name	RT	RI_*Cal*_	RI_*Lit*_	Clonal lines								Check varieties			
					CSIR-IHBT-RD-01		CSIR-IHBT-RD-02		CSIR-IHBT-RD-03		CSIR-IHBT-RD-04		Himroz		Jwala	
					2021	2022	2021	2022	2021	2022	2021	2022	2021	2022	2021	2022
Oxygenated monoterpenes																
1	*α*-Pinene	5.38	943	942	*nd*	*nd*	0.98	0.26	0.35	0.46	0.78	1.33	0.83	0.53	0.49	0.54
2	*β*-Myrcene	6.771	992	998	*nd*	*nd*	0.25	0.23	*nd*	*nd*	0.43	0.15	0.47	0.38	0.34	0.34
3	Linalool L	10.355	1099	1095	*nd*	*nd*	0.71	0.84	0.38	1.00	*nd*	*nd*	*nd*	*nd*	*nd*	*nd*
4	*cis*-Rose oxide	10.718	1110	1106	0.26	0.25	1.15	0.36	1.04	0.34	1.65	0.25	1.67	1.16	1.16	1.14
5	*trans*- Rose oxide	11.298	1128	1122	0.14	0.13	0.29	0.18	0.29	0.15	1.23	0.1	1.26	1.43	1.13	1.34
6	Terpinen-4-ol	13.266	1182	1174	*nd*	*nd*	*nd*	0.22	0.14	0.21	*nd*	*nd*	*nd*	*nd*	*nd*	*nd*
7	*α*-Terpineol	13.785	1195	1188	*nd*	*nd*	0.35	*nd*	0.25	0.16	0.37	0.31	0.34	0.29	0.27	0.28
8	Citronellol	15.279	1239	1228	20.57	20.35	27.97	27.37	30.18	34.25	37.20	44.75	36.95	34.85	35.73	34.89
9	Neral	15.347	1241	1238	0.27	0.26	*nd*	*nd*	*nd*	*nd*	*nd*	*nd*	0.59	1.08	1.10	1.08
10	Geraniol	16.319	1268	1256	15.69	15.63	23.72	20.55	23.17	24.93	27.41	23.16	27.22	27.75	28.03	27.76
	Sub total				36.93	36.62	55.42	50.01	55.8	61.5	69.07	70.05	69.33	67.47	68.25	67.37
Oxygenated sesquiterpenes																
11	Citronellyl acetate	19.161	1348	1352	0.50	0.50	0.72	0.76	0.82	0.72	0.34	0.77	0.32	0.32	0.30	0.32
12	Eugenol	19.302	1352	1356	0.21	0.20	1.72	1.03	2.18	1.17	1.08	1.81	0.99	0.77	0.67	0.80
13	Neryl acetate	20.141	1376	1365	2.49	2.41	2.76	2.26	2.33	2.35	1.65	1.74	1.01	1.12	1.14	1.13
14	Methyleugenol	20.894	1396	1400	0.36	0.37	1.47	0.91	1.24	0.55	0.75	0.81	0.77	0.71	0.69	0.72
	Sub total				3.56	3.48	6.67	4.96	6.57	4.79	3.82	5.13	3.09	2.92	2.8	2.97
Sesquiterpenes hydrocarbons																
15	trans-Caryophyllene	21.655	1420	1419	0.45	0.43	1.26	0.72	0.61	0.53	0.64	0.41	0.67	0.53	0.49	0.53
16	*α*-Guaiene	22.153	1435	1439	0.35	0.33	0.74	0.48	*nd*	*nd*	0.57	0.31	0.56	0.5	0.48	0.48
17	*α*-Humulene	22.841	1456	1456	0.41	0.42	0.87	1.60	0.45	0.43	0.60	0.34	0.58	0.57	0.57	0.58
18	Germacrene D	23.672	1481	1482	0.80	0.77	2.50	0.27	1.37	1.21	1.23	1.13	1.00	0.88	0.88	0.89
19	Pentadecane	24.231	1497	1500	0.38	0.39	0.29	0.27	0.37	0.29	0.28	0.36	0.28	0.27	0.27	0.27
20	Farnesene	24.341	1502	1509	0.28	0.29	0.74	0.42	0.48	0.36	0.44	0.39	0.45	0.38	0.35	0.37
	Sub Total				2.67	2.63	6.40	3.76	3.28	2.82	3.76	2.94	3.54	3.13	3.04	3.12
Aliphatic hydrocarbons																
21	Heptadecane	30.327	1696	1700	2.45	2.44	1.59	1.71	2.19	1.62	1.48	1.31	1.51	1.24	1.40	1.38
22	*n*-Octadecane	33.140	1802	1800	1.66	0.38	0.19	0.21	0.22	0.16	*nd*	*nd*	*nd*	*nd*	*nd*	*nd*
23	9-Eicosene, (*E*)-	35.132	1872	1874	4.33	4.35	2.26	3.31	3.22	3.09	1.88	1.77	1.87	1.72	1.72	1.73
24	*n*-Nonadecane	35.843	1896	1900	28.54	28.78	16.76	19.85	18.4	15.47	13.02	10.83	13.12	13.7	14.20	13.73
25	Eicosane	38.389	2001	2000	3.00	3.04	1.52	2.36	1.45	1.58	1.12	1.07	1.12	1.22	1.22	1.22
26	Heneicosane	40.848	2095	2100	15.70	15.80	8.66	12.63	7.86	7.69	5.33	4.96	5.37	5.64	5.81	5.63
	Sub total				55.68	54.79	30.98	40.07	33.34	29.61	22.83	19.94	22.99	23.52	24.35	23.69
	Total				98.84	97.52	99.47	98.80	98.99	98.72	99.48	98.06	98.95	97.04	98.44	97.15
	*C/G Ratio				1.31	1.30	1.18	1.33	1.30	1.37	1.36	1.93	1.36	1.26	1.27	1.26

RT; Retention time RI_*Cal*_; Retention index calculated*,* RI_*Lit*_; Retention index from literature, *nd* = not detected, *C/G Ratio = Citronellol/Geraniol ratio.

Diverse chemotypic distinctions were obtained in the essential oil composition. The *α*-pinene (0.26–1.33%) was detected in all the samples except clonal selection CSIR-IHBT-RD-01 for both the experimental years 2021 and 2022. Likewise, the *β-*myrcene (0.15–0.47%) was detected in all the samples studied except for the clonal selections CSIR-IHBT-RD-01 and CSIR-IHBT-RD-03 for both years. The linalool L (0.38–1.00%) was detected in clonal selection CSIR-IHBT-RD-02 and CSIR-IHBT-RD- 03. It was absent in other clonal selections, including check varieties Himroz and Jwala. The terpinen-4-ol (0.14–0.22%) was detected in CSIR-IHBT-RD-03 and CSIR-IHBT-RD-02 during the first year, while it was detected only in CSIR-IHBT-RD-02 during the second year. It was absent in other clonal lines, including check varieties. The *α*-terpineol (0.16–0.37%) was detected in all the clonal selections, including check varieties except CSIR-IHBT-RD-01 for both years. Similarly, the essential oil compound neral (0.26–1.10%) was detected in clonal selection CSIR-IHBT-RD-01 and check varieties Himroz and Jwala during both the experimental years. Neral was not detected in the samples of CSIR-IHBT-RD-02, CSIR-IHBT-RD-03 and CSIR-IHBT-RD-04. The *α*-Guaiene (0.31–0.74%) was detected in all the clonal selections and check varieties except CSIR-IHBT-RD- 03 during both years. The *n*-Octadecane (0.16 to 1.66%) was detected in all clonal selections except CSIR-IHBT-RD-04, including check varieties Himroz and Jwala during 2021 and 2022.

Eighteen compounds were present in all the clonal lines and check varieties studied (Table 5). Significant variations were observed based on a t-test using standard deviation for essential oil components among the clonal lines and check varieties for both years. Based on the mean value of the component, the highest content of *cis*-rose oxide (1.67%) and trans-rose oxide (1.26%) were observed for check variety Himroz during 2021 that was statistically at par with the clonal selection CSIR-IHBT-RD-04. However, during 2022 significant variations were observed for check varieties Himroz (1.16 and 1.43%) and Jwala (1.14 and 1.34%) for *cis*-rose and trans-rose oxides, respectively.

**Table 5:** Variations of essential oil compounds in clonal lines and two check varieties of damask rose based on “t-test” using pooled standard deviation for volatile oil components.

S. No	Compound name	Clonal lines								Check varieties			
		CSIR-IHBT-RD-01		CSIR-IHBT-RD-02		CSIR-IHBT-RD-03		CSIR-IHBT-RD-04		Himroz		Jwala	
		2021	2022	2021	2022	2021	2022	2021	2022	2021	2022	2021	2022
1	*cis*-Rose oxide	0.26	0.25	1.15	0.36	1.04	0.34	1.65	0.25	1.67	1.16*	1.16	1.14*
2	*trans*-Rose oxide	0.14	0.13	0.29	0.18	0.29	0.15	1.23	0.10	1.26	1.43*	1.13	1.34*
3	Citronellol	20.57	20.35	27.97	27.37	30.18	34.25	37.20	44.75*	36.95	34.85	35.73	34.89
4	Geraniol	15.69	15.63	23.72	20.55	23.17	24.93	27.41	23.16	27.22	27.75	28.03*	27.76
5	Citronellyl acetate	0.50	0.50	0.72	0.76	0.82*	0.72	0.34	0.77	0.32	0.32	0.30	0.32
6	Eugenol	0.21	0.20	1.72	1.03	2.18*	1.17	1.08	1.81*	0.99	0.77	0.67	0.80
7	Neryl acetate	2.49	2.41	2.76*	2.26	2.33	2.35	1.65	1.74	1.01	1.12	1.14	1.13
8	Methyleugenol	0.36	0.37	1.47*	0.91*	1.24	0.55	0.75	0.81	0.77	0.71	0.69	0.72
9	trans-Caryophyllene	0.45	0.43	1.26*	0.72*	0.61	0.53	0.64	0.41	0.67	0.53	0.49	0.53
10	α-Humulene	0.41	0.42	0.87*	1.60*	0.45	0.43	0.60	0.34	0.58	0.57	0.57	0.58
11	Germacrene D	0.80	0.77	2.50*	0.27	1.37	1.21*	1.23	1.13	1.00	0.88	0.88	0.89
12	Pentadecane	0.38*	0.39	0.29	0.27	0.37*	0.29	0.28	0.36*	0.28	0.27	0.27	0.27
13	Farnesene	0.28	0.29	0.74*	0.42*	0.48	0.36	0.44	0.39	0.45	0.38	0.35	0.37
14	Heptadecane	2.45*	2.44*	1.59	1.71	2.19	1.62	1.48	1.31	1.51	1.24	1.40	1.38
15	9-Eicosene, (E)-	4.33*	4.35*	2.26	3.31	3.22	3.09	1.88	1.77	1.87	1.72	1.72	1.73
16	n-Nonadecane	28.54*	28.78*	16.76	19.85	18.40	15.47	13.02	10.83	13.12	13.7	14.20	13.73
17	Eicosane	3.00*	3.04	1.52	2.36	1.45	1.58	1.12	1.07	1.12	1.22	1.22	1.22
18	Heneicosane	15.70*	15.8*	8.66	12.63	7.86	7.69	5.33	4.96	5.37	5.64	5.81	5.63
	C/G Ratio	1.31	1.30	1.18	1.33	1.30	1.37	1.36	1.93*	1.36	1.26	1.27	1.26

*Significant at *P* < 0.05 (t-tab. = 2.27), *#*C/G Ratio is citronellol/geraniol ratio.

The highest citronellol content, *i.e.,* 37.20 and 44.75%, were observed for clonal selection CSIR-IHBT-RD-04 for 2021 and 2022, respectively. Clonal line CSIR-IHBT-RD-04 had the highest citronellol content in the essential oil compared with other clonal lines during both years. It was significantly higher than Himroz and Jwala in 2022. The geraniol content was significantly high for check variety, Jwala (28.03%), during 2021 compared to the mean of all the clonal lines, but statistically, it was at par with CSIR-IHBT-RD-04 and Himroz. The clonal selection CSIR-IHBT-RD-01 was significantly inferior for geraniol content in both years compared to the mean value of the clone. Likewise, the essential oil compound citronellyl acetate (0.82%) was statistically significant for clonal selection CSIR-IHBT-RD-03 in 2021. However, non-significant variations have been observed for all the clonal lines and check varieties during 2022. The eugenol content (2.18% and 1.81%) was statistically significant for clonal selection CSIR-IHBT-RD-03 and CSIIR-IHBT-RD-04 during 2021 and 2022, respectively. The neryl acetate (2.76%) was statistically significant for clonal selection CSIR-IHBT-RD-02 during 2021 but was at par with other clones in 2022. The essential oil compounds such as methyleugenol (1.47% and 0.91%), *trans*-caryophyllene (1.26% and 0.72%) and *α*-humulene (0.87% and 1.60%) were observed to be significant for clonal selection CSIR-IHBT-RD-02 during the first and second year, respectively. Similarly, germacrene D content (2.50% and 1.21%) was statistically significant for CSIR-IHBT-RD-02 and CSIR-IHBT-RD-03 during the first and second years, respectively. The content of pentadecane (0.38% and 0.37%) was statistically significant for CSIR-IHBT-RD-01 and CSIR-IHBT-RD-03 during the first year but was non-significant in all clones and check varieties except CSIR-IHBT-RD-04 (0.36%) during the second year. Farnesene content (0.74% and 0.42%) was statistically significant for the clonal selection CSIR-IHBT-RD-02 for both years. The essential oil compounds such as heptadecane (2.45% and 2.44%), 9-eicosene-E (4.33% and 4.35%) and n-nonadecane (28.54% and 28.78%) were statistically significant for clonal selection CSIR-IHBT-RD-01 during 2021 and 2022, respectively. Eicosane content (3.0%) was statistically significant for clonal selection CSIR-IHBT-RD-01 during 2021 but was statistically non-significant during 2022. The heneicosane content (15.7% and 15.8%) was statistically significant for clonal selection CSIR-IHBT-RD-01 during 2021 and 2022, respectively. The citronellol/geraniol ratio (C/G ratio) was found statistically significant for clonal selection CSIR-IHBT-RD-04 in 2022.

Based on the concentration of chemical compounds included in the international standards^34^ for the essential oil of rose (*Rosa* × *damascena* Miller), the percentage of citronellol (20.35–44.75%), geraniol (15.63–28.03%) and heptadecane (1.24 to 2.45%) were found at permissible range in all the samples studied. The amount of n-nonadecane was consistent with international standards in clonal selection CSIR-IHBT-RD-03, CSIR-IHBT-RD-04, Himroz and Jwala (10.83–18.40%), while it was higher in CSIR-IHBT-RD-01 and CSIR-IHBT-RD-02 and varies from 16.76 to 28.78% during both the year. The Heneicosane percentage was higher (5.33–15.8%) than the international standards (1.5 to 5.5%) in all the samples studied except CSIR-IHBT-RD-04.

**Figure 8:**
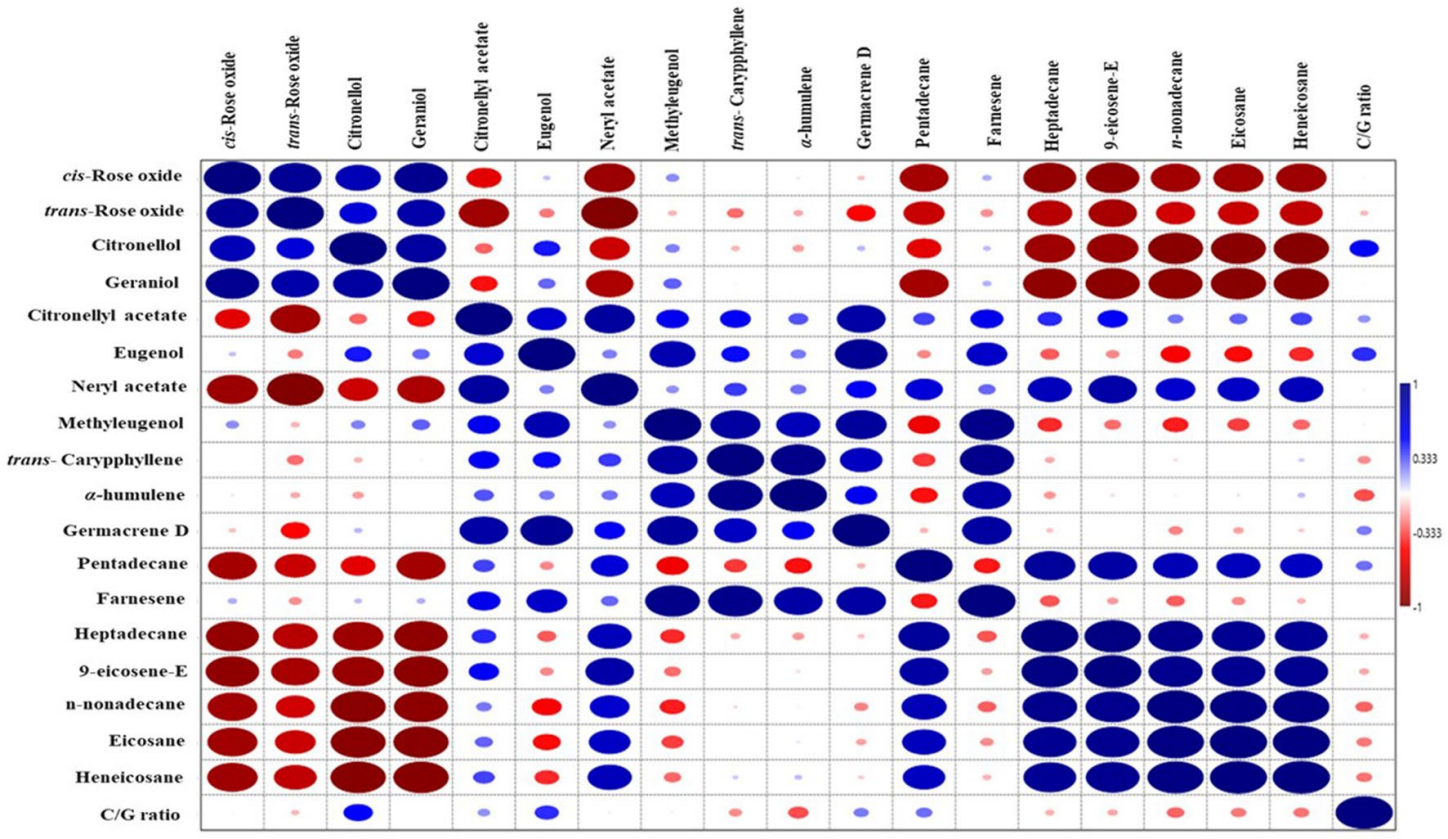
Correlation studies among the essential oil compounds based on pearson correlation matrix (pooled data of 2021 and 2022).

Correlation studies were performed to study the association between essential oil compounds using the pooled mean value of two years (Fig. 8). Under the oxygenated monoterpenes group, significant correlations of* cis*-rose oxide were obtained with *trans*-rose oxide (r = 0.91) and geraniol (r = 0.92), respectively. It showed significant negative correlation with neryl acetate, pentadecane, heptadecane, 9-eicosene-E, n-nonadecane, eicosane and heneicosane (r =  − 0.83 to − 0.92.). The *trans*-rose oxide had a significant negative correlation with neryl acetate (r =  − 0.98), citronellyl acetate (r =  − 0.84) and 9-eicosene-E (r =  − 0.82). The citronellol had a positive and significant correlation with geraniol (r = 0.87). It has significant negative correlation with heptadecane, 9-eicosene-E, n-nonadecane, eicosane and heneicosane (r =  − 0.87 to − 0.96). Geraniol had significant negative correlation with pentadecane, heptadecane, 9-eicosene-E, n-nonadecane, eicosane and heneicosane (r =  − 0.83 to − 0.96).

In the oxygenated sesquiterpenes group, citronellyl acetate positively correlated with neryl acetate (r = 0.84) and germacrene D (r = 0.83). Eugenol significantly correlated with germacrene D (r = 0.91). Neryl acetate had a significant positive correlation with 9-eicosene-E (r = 0.82). The methyleugenol had a significant positive correlation with trans-caryophyllene (r = 0.87) and germacrene D (r = 0.90), whereas a highly positive and significant correlation has been observed with farnesene (r = 0.96). In the sesquiterpenes hydrocarbons group**,** trans-caryophyllene had a significant positive correlation with *α*-humulene (r = 0.96) and farnesene (r = 0.94). The* α*-humulene and germacrene D had a significant positive correlation with farnesene (r = 0.85). Pentadecane had significant positive correlation with heptadecane (r = 0.88) and 9-eicosene-E (r = 0.83). Similarly, under aliphatic hydrocarbons group, heptadecane had a significant positive correlation with 9-eicosene-E, n-nonadecane, eicosane and heneicosane (r = 0.91 to 0.98). 9-eicosene-E had significant positive correlation with n-nonadecane, eicosane and heneicosane (r = 0.93 to 0.94). Likewise, n-nonadecane significantly correlated with eicosane (r = 0.99) and heneicosane (r = 0.98). Eicosane had significant positive correlation with heneicosane (r = 0.99).

The variations in essential oil composition observed in the present study are possibly due to the genotypic response of different selections to changing weather conditions during the flowering period for both years. The meteorological conditions, such as the maximum temperature, minimum temperature, and relative humidity at the evening, were comparatively higher during 2022. In contrast, total rainfall, relative humidity at the morning and sunshine hours were comparatively higher during 2021. The clonal selection CSIR-IHBT-RD-04 exhibited significantly higher citronellol, eugenol and pentadecane content, including citronellol/geraniol ratio (C/G ratio) 2022 compared to 2021. Compared to other lines, a positive response of CSIR-IHBT-RD-04 for citronellol content in essential oil was obtained at a relatively high-temperature regime (upto 30 °C in 2022, compared to 26.50 °C in 2021) and dry climate (68.35 mm rainfall and 48.15% relative humidity in 2022, compared to 108 mm rainfall and 62.0% relative humidity in 2021) during the flowering period. A similar type of difference in essential oil compounds has earlier been observed, confirming the influence of ecological and environmental conditions^35^, genetic factors^36^ and post-harvesting on the biosynthesis of secondary metabolites.

Our results for major essential oil compounds of damask rose align with the previous reports where the acyclic monoterpene alcohols (citronellol and geraniol) and long-chain hydrocarbons (n-nonadecane and heneicosane) were the major components^1,37^. The acyclic monoterpene alcohol, i.e., citronellol, is responsible for the rose-like aroma of the essential oil^1,38^. A higher amount of citronellol in the essential oil indicates higher quality^1^. Earlier studies reported the highest amount of 42% citronellol in the essential oil of damask rose from the western Himalayan conditions^39^. The most important/sensitive indicator of damask rose oil odor quality is the citronellol/geraniol ratio (C/G ratio) between 1.25 and 1.30^1,38^. In our present study, the C/G ratio for essential oil samples varies from 1.18 to 1.93%. The clonal selection CSIR-IHBT-RD-04 was superior in flower yield and flower frequency/plant/day compared to other clonal lines. The essential oil content was higher in CSIR-IHBT-RD-04 during both years compared to other clonal lines except for check variety Himroz. Based on the GC–MS profiling of the essential oil, CSIR-IHBT-RD-04 captures unique chemotypic diversity in terms of the highest citronellol content (37.20% in 2021 and 44.75% in 2022). The C/G ratio was also significantly higher in CSIR-IHBT-RD-04 during 2022. The clonal line CSIR-IHBT-RD-04 has also been registered with the Indian Council of Agricultural Research-Plant Germplasm Registration Committee, New Delhi, under accession number IC0635435, INGR20105 as new germplasm based on its peculiar characteristics.

## Conclusion

The present study investigated the variations for floral traits, the essential oil profile of the four clonal lines, and two check varieties of damask rose. The study was undertaken to identify superior clonal selection for high yield and quality oil composition. The selection CSIR-IHBT-RD-04 was superior in flower yield and had higher flower frequency/plant/day than other clonal lines. The essential oil content was also higher in CSIR-IHBT-RD-04 when compared with other clonal lines except for check variety Himroz. Based on GC- MS profiling of essential oil, CSIR-IHBT-RD-04 displays unique chemotypic diversity in terms of the highest citronellol content and citronellol/geraniol (C/G) ratio, which is the chief indicator of high quality. Clonal selection CSIR-IHBT-RD-04 may be used as a parental line in the hybridization program for genetic improvement of damask rose.

## Data Availability

All the data associated is within the manuscript.
